# Coherent and incoherent scatterings for measurement of mandibular bone density and stable iodine content of tissue

**DOI:** 10.4103/0971-6203.54855

**Published:** 2009

**Authors:** Amandeep Sharma, Mohinderpal Singh, Bhajan Singh, Balvir S. Sandhu

**Affiliations:** Department of Physics, Punjabi University, Patiala-147002, India

**Keywords:** Coherent and incoherent scatterings, intensity ratio, phantom

## Abstract

The aim of present study is to investigate the feasibility of gamma ray scattering for measurements of mandibular bone density and stable iodine content of tissue. Scattered spectra from solutions of K_2_HPO_4_ in distilled water (a phantom simulating the mandibular bone) and KI in distilled water filled in a thin plastic vial (a phantom simulating the kinetics of thyroid iodine) are recorded for 59.54 and 145 keV incident gamma rays, respectively. A high-purity germanium detector is placed at various angular positions to record the scattered spectra originating from interactions of incident gamma rays with the phantom. The measured intensity ratio of coherent to incoherent scattered gamma rays, corrected for photo-peak efficiency of HPGe detector, absorption of gamma rays in air column present between phantom and detector, and self-absorption in the phantom, is found to be increasing linearly with increase in concentration of K_2_HPO_4_ and KI in distilled water within experimental estimated error of <6%. The regression lines, obtained from experimental data for intensity ratio, provide the bone density and stable iodine contents of thyroid. The present non-destructive technique has the potential for a measure of mandibular bone density and stable iodine contents of thyroid.

## Introduction

Metabolic bone diseases such as osteoporosis, a bone disease characterized by progressive loss of bone mass often resulting in fracture after minimal trauma, lead to loss of bone minerals from both compact and trabecular bones, which eventually leaves the affected bone vulnerable to traumatic fracture. Osteoporosis causes significant mortality and loss of quality of life. The bone mineral density of the mandible is quite useful to study bone resorption after tooth loss and to determine the relationship between mandibular and skeletal bone mineral density. An accurate estimation of the bone density, an indicator for determining osteoporosis and for follow-up study of the patient under the therapy for osteoporosis, is required. In this context, various bone density-measuring methods are available and coherent to incoherent scattering ratio method is a typical one. Medical diagnostic techniques based on gamma rays scattering are relatively new and many are still under development. The combined use of coherent and incoherent scatterings at gamma ray energies < 100 keV has been proven quite useful, particularly in relation to osteoporosis.

Taking KI saturates the thyroid gland with stable (non-radioactive) iodine. This also prevents or reduces the amount of radioiodine that can be taken up by the thyroid. An accurate method for the in vivo determination of stable iodine concentration of tissues during kinetic studies of radiographic substances containing iodine is required. The currently used non-destructive methods, based on the detection of X-ray fluorescent photons, suffer from the limitation due to interfering effect of attenuation of radiation in tissue layers overlying the site to be measured.

The coherent to incoherent scattering intensity ratio method has been quite successful in various fundamental and medical applications of gamma radiations. Morgan *et al*.[1,2] have applied this technique to measure mandibular bone density by observing the scattered spectrum from solutions of K_2_HPO_4_ in distilled water, a phantom simulating the mandibular bone, but the work is limited to scattering angle of 150° only. They have also applied this technique for fat fraction[3] measurement. Puumalainen *et al*.[4] have successfully applied this technique for the qualitative assessment of soft-tissue iodine content, but the study is limited to scattering of 59.54 keV gamma photons at scattering angles of 45° and 90°. More recently, Singh *et al*.[5,6] have used this non-destructive technique for assigning effective atomic number to composite materials of industrial and scientific interest.

In the present study, measurements are made to study the variation of Rayleigh to Compton scattered intensity as a function of K_2_HPO_4_ and KI concentration in water (simulating the mandibular bone density and kinetics of thyroid iodine respectively) for 59.54 and 145 keV incident gamma rays, respectively. The scattered gamma rays are detected by a high-resolution HPGe semiconductor detector. The experiments are performed for different concentrations of K_2_HPO_4_ and KI in distilled water. The observed intensity ratio of Rayleigh to Compton scattered gamma flux, corrected for photo-peak efficiency of the HPGe gamma detector and absorption of photons in the target and air is determined for various concentrations. The results of our experiments are in good agreement with that of Morgan *et al*.[1,2] and Puumalainen et al.[4] with regard to 59.54 keV incident gamma rays.

## Theory of Coherent and Incoherent Scatterings

Theoretically, each gamma ray interaction process could be used as the basis for a noninvasive assessment of mandibular bone density and the stable iodine content of tissue. In scattering experiments, for a gamma ray flux impinging on a target (phantom in the present study), there is significant probability for coherent (Rayleigh) scattering to occur in addition to well-known incoherent (Compton) scattering. In the observed scattered spectra originating from interactions of primary gamma ray flux with the phantom under study, there are two peaks in the observed scattered spectra, a full-energy peak resulting from coherently scattered gamma rays and other a broader peak of lower energy representing secondary gamma rays that have suffered energy loss after scattering in the target material, and are referred to as Rayleigh (coherent or elastic) and Compton (incoherent or inelastic) peaks, respectively, in gamma ray spectroscopy.

Rayleigh (or elastic) scattering is the process in which the scattered gamma ray has the same energy as that of incident gamma ray, and is predominant at low incident gamma ray energies, small scattering angles and high atomic number of the target. Theoretically, the probability for occurrence of Rayleigh scattering is given as:

1 $$\frac{{\mathrm{d\sigma}}_{R}}{\mathrm{d\Omega}}=\frac{{r_{0}}^{2}}{2}(1+\cos^{2}\theta )|F(q,Z){|}^{2}$$

Where F (q, Z) is the atomic form factor.

The Compton peak has energy (E_c_) less than incident gamma ray energy (E_o_) and its FWHM (Full width at half the maximum height) is larger than a photo-peak at the same energy (E_c_) mainly because of angular aperture of the spectrometer, multiple scattering in the target and the so-called Compton profile.[7] The energy of Compton scattered gamma ray is uniquely related to the incident gamma ray energy (E_o_) and scattering angle (θ), and is given as

2 $${\text{E}}_{\text{c}}\text{ = }\frac{{\text{E}}_{0}}{1+\text{α }(1-\cos\text{θ})}$$

Where α is gamma ray energy in units of rest mass energy of electron.

The probability for occurrence of Compton scattering at an angle θ is provided by well-known Klein-Nishina relation given below

3 $$\frac{d\sigma_{\mathrm{KN}}}{d\Omega }=\frac{{r_{0}}^{2}}{2}{\left[\frac{1}{1+\alpha (1-\cos\theta )}\right]}^{2}[1+\cos^{2}\theta +\frac{[\alpha (1-\cos\theta ){]}^{2}}{1+\alpha (1-\cos\theta )}]$$

Where r_o_ (=2.8179 × 10^-15^ m) is the classical electron radius. Scattering from bound electrons is calculated as

4 $$\frac{\text{d}\sigma_{\text{comp}}}{\text{d}\Omega }\text{ = }\frac{\text{d}\sigma_{\text{KN}}}{\text{d}\Omega }\text{S(q,Z)}$$

Where S (q, Z) is the incoherent scattering function, with q representing the momentum transferred to the electron and Z is the atomic number of the target.

The Rayleigh to Compton scattering cross-section ratio becomes

5 $$\text{R = }\frac{\text{d}\sigma_{\text{R}}}{\text{d}\Omega_{\text{comp}}}\text{ α }\frac{|{\text{F(q,Z)|}}^{2}}{\text{S(q,Z)}}$$

Rayleigh to Compton scattering ratio has a power relation to Z in the region of elemental interest, and this power dependence is based on the ratio F^2^/S. Hubbell *et al*.[8] have provided theoretical data for calculation of Rayleigh to Compton cross-section ratio from the parameters for F(q, Z) and S(q, Z).

## Experimental Set-Up of Present Work

In scattering experiments, for a gamma ray flux impinging on a target, there is significant probability for Rayleigh scattering to occur in addition to well-known Compton scattering. The principle of present measurements is to observe simultaneously the intensities of Rayleigh and Compton scattered gamma rays at a particular scattering angle using a high-resolution semiconductor detector. While designing an experiment based on this technique, care must be taken to select properly the incident gamma ray energy, scattering angle and material to be probed. The intensity of coherent and incoherent peaks should be sufficient, and the two peaks must be well separated from each other, otherwise the analysis procedure becomes complicated. Because this method involves the ratio of Rayleigh to Compton peaks, the cross-section ratio depends on the Z-number of the material with which primary gamma ray flux has to interact, for a fixed geometrical source-sample-detector arrangement and incident gamma ray energy.

**Figure 1:**
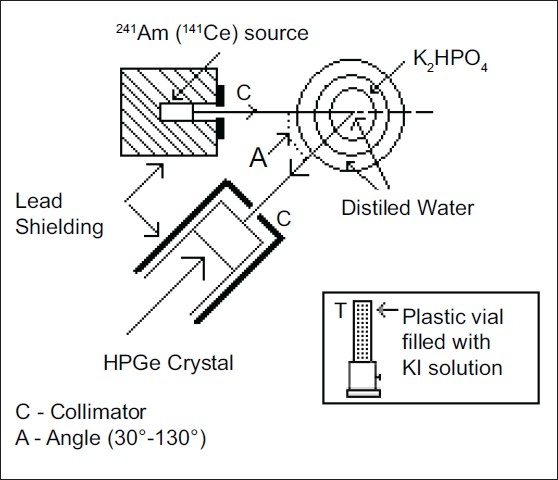
Experimental set-up

Two separate experiments, one for madibular bone density using 59.54 keV gamma rays and the other for stable iodine content of tissue using 145 keV gamma rays, are performed simultaneously. Figure 1 shows narrow beam geometry used in the present measurements to observe simultaneously the coherent and incoherent scattered gamma ray flux. A well-collimated beam of 59.54 keV gamma rays from ^241^Am radioactive source (strength 7.4 GBq, T_1/2_ = 458 years) irradiates the phantom simulating mandibular bone. This source is chosen because its low gamma ray energy favours coherent scattering and at the same time this energy is not so low as to cause severe gamma ray losses due to phantom (simulating mandibular bone) attenuation. ^241^Am does not emit other photons in the region of 59.54 keV or above; thus, coherent and other peaks can be distinguished without interference from other emissions. In addition, ^241^Am has a long half-life and it is readily available. Bone phantoms are prepared using K_2_HPO_4_ and distilled water. Aqueous solutions of K_2_HPO_4_ in the concentration range 0-30 g in steps of 6 g in 100 mL of water are used to simulate the bone density ranging from 1000 to 1200 kg/m^3^. The solutions are filled in plastic containers (diameter 43, 65 and 82 mm for central, middle and outer containers, respectively) positioned at the center of rotation of the source and detector assemblies. For measurement of iodine content of tissue, a well-collimated beam of 145 keV gamma rays from ^141^Ce source of strength 0.74 GBq irradiates the phantom. The sample (phantom) is in the form of KI of different amounts ranging from 2 to 10 g in step of 2 g in 10 mL of distilled water filled in a plastic vial (10 mm diameter) kept in a stand with screw to adjust its height.

A high-purity germanium detector (HPGe) is housed in a lead castle similar to one used for the source, and is collimated in an identical manner. The scattered gamma rays from the phantom (simulating mandibular bone) are detected by a high-resolution HPGe semiconductor detector (45.5 mm diameter and 46.1 mm length, and 75 cm^3^ volume) placed at scattering angles of 90°, 110°, 130° and 150° to the primary incident gamma ray beam. For thyroid iodine measurement, the scattered gamma rays from phantom are detected by an HPGe solid-state detector (56.4 mm diameter and 29.5 mm length, and 75 cm^3^ volume) placed at three different scattering angles of 50°, 70° and 90° relative to the incident gamma ray beam. The HPGe detector is properly shielded by a cylindrical lead shielding having the inner side covered with 2-mm thick iron and 1-mm thick aluminium, with iron facing lead to absorb x-rays emitted by lead shielding. The distances of center of phantom under study from the source and detector collimators (hole radius of each is 4 mm) are kept 120 mm (100 mm for iodine content measurements) and 100 mm, respectively, so that the angular spread about the median ray in the direction of gamma ray detector is limited to ±2.3° (±2.9° for thyroid iodine measurements). The axes of HPGe detector, source and cylindrical collimators pass through the center of phantom. The field of view of HPGe detector is confined to phantom only. It has been verified that radiation scattered from the source collimator opening do not reach directly the active volume of HPGe detector, and the background near the detector assembly comes to natural background level in the laboratory when source window is closed.

In the present measurements, the Canberra HPGe detector and electronic modules (power supply and amplifier) are used, and the experimental data are accumulated on a PC-based ORTEC Mastreo-32 Multichannel analyser (MCA). The observed scattered spectra provide Rayleigh to Compton intensity ratio, for each angular position of the detector, for each of the solutions simulating mandibular bone/kinetics of thyroid iodine.

## Experimental Measurements

The properly shielded HPGe detector is placed at the desired angular position relative to the primary incident gamma ray beam. The spectrometer is calibrated using standard calibration gamma-ray sources of known energy. The following procedure is adopted for the present measurements.

1. The phantom-in scattered spectra are recorded for a period of 5 ks by placing each phantom, different concentrations of K_2_HPO_4_ (or KI solution in thin plastic vial), in the primary gamma-ray beam. The registered events originate from interactions in the phantom and background events.
2. The background is recorded for the same duration after removing the solution from the containers (or vial) to permit the registration of events due to cosmic rays and to any other process independent of the phantom.

The measurements for different concentrations of solutions are performed in the above sequence to minimize the effect of any possible drift in the system. Moreover, the calibration and stability of the system are checked before and after a recording of each of the scattered spectra, and adjustments are made, if required. The subtraction of events recorded under condition (ii), from those under condition (i) results in events originating from interaction of gamma rays with the phantom.

**Figure 2:**
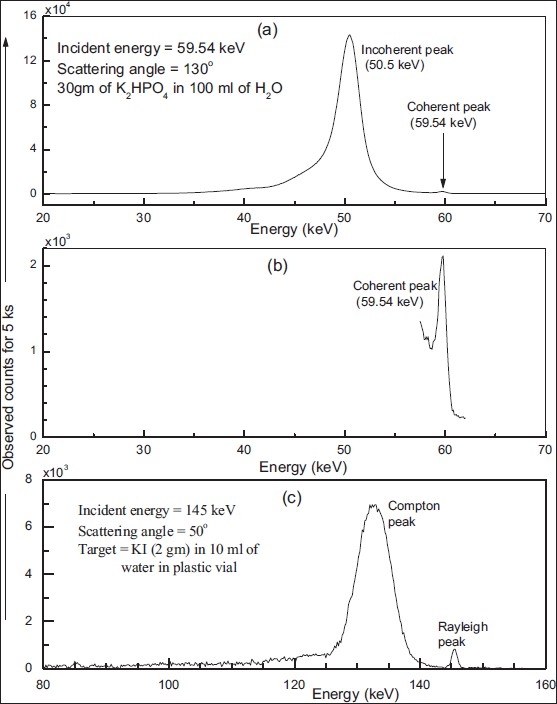
(a) A typical observed spectra at a scattering angle of 130° for a phantom (K_2_HPO_4_ concentration 30 g in 100 mL) when irradiated by 59.54 keV incident photons for 5 ks duration, (b) zoom in spectra of region of coherent peak, and (c) a typical observed spectra at a scattering angle of 50° for a phantom (KI concentration, 2 g in 10 mL of water) when irradiated by 145 keV incident gamma rays

A typical observed spectrum, corrected for background events, from phantom (30 g of K_2_HPO_4_ in 100 mL distilled water) when irradiated by 59.54 keV gamma rays (at scattering angle of 130°) is shown in [Figure 2a]. The coherent and incoherent peaks are observed at 59.54 and 50.5 keV, respectively. The intensity of coherent peak is much smaller in comparison to incoherent peak owing to low effective atomic number of the phantom and lesser probability for coherent process to occur at large scattering angles. [Figure 2b] shows zoom in the spectrum of region of coherent peak [Figure 2a] showing its presence, which otherwise is not clearly visible because of observed higher counting rate under the incoherent peak. [Figure 2c] shows a typical observed spectrum, corrected for background events, from phantom (2 g of KI in 10 mL distilled water) when irradiated by 145 keV gamma rays (at scattering angle of 50°). The Rayleigh and Compton peaks are clearly visible at energies of 145 and 131.6 keV, and distinguished from each other in the scattered spectra. The intensity of Rayleigh peak is considerably smaller in comparison to Compton peak owing to lesser probability for Rayleigh process to occur in comparison to Compton scattering at the chosen scattering angle for a given incident energy. The observed spread in the coherent peak is caused by inherent energy resolution of the HPGe detector. The spread in the observed incoherent peak is caused by finite angular aperture of the source and detector collimators, Doppler broadening of incoherent peak and intrinsic energy resolution of the HPGe detector.

In the scattered spectra, Rayleigh and Compton peaks are not simple but are with superimposed continuum; hence, the following formula[9] to subtract the additional unwanted counts included in this process has been used.

6 $$\text{Peak Area=}\sum_{i=A}^{B}C_{i}-(\text{B}-\text{A)}\frac{{\text{C}}_{\text{A}}+{\text{C}}_{\text{B}}}{2}$$

Where A and B are the channel numbers specifying the region of observed photo-peak, C_A_ and C_B_ are the counts at the respective channels and C_i_ is the simple integration of counts between the limits A to B. The intensity ratios of coherent to incoherent scattered peaks are corrected for photo-peak efficiency of the HPGe detector, absorption of gamma rays in air column present between phantom and HPGe detector, and self-absorption in the phantom. The actual counts for each peak of phantom, simulating madibular bone, are calculated using the following equation.

7 $${\text{N}}_{actual}=\frac{{\text{N}}_{observed}}{\beta_{\gamma 1\alpha 1}\beta_{\gamma 1\varpi 1}\beta_{\gamma 1t}\beta_{\gamma 2\varpi 2}\beta_{\gamma 2t}\beta_{\gamma 2\varpi 2}\beta_{\gamma 2\alpha 2}\varepsilon_{\gamma 2}}$$

Where N_observed_ is the observed intensity under the coherent (or incoherent) peak. β_γ1a1_, β_γ1ω1_, β_γ1*t*_, are the correction factors for absorption of incident gamma rays in the air column between source and phantom, outer water column and K_2_ HPO_4_ solution column, respectively. β_γ2ω2_, is the correction factor for scattered photons in central water column. β_γ2*t*_, β_γ2ω2_, β_γ2a2_ are the correction factors for absorption of scattered gamma rays in K_2_ HPO_4_ solution column, outer water column, air column between phantom and detector, respectively. ɛ_γ2_ is the photo-peak efficiency of HPGe detector for scattered coherent (or incoherent) gamma rays.

The intensities under these peaks of phantom, simulating thyroid iodine, are deduced from the recorded scattered spectra, as per relation[6] given below:

8 $${\text{N}}_{\text{actual}}=\frac{{\text{N}}_{\text{obs}}}{{\text{ε}}_{\text{γ}}{\text{β}}_{\text{γa}}{\text{β}}_{\text{γt}}}$$

Here N_obs_ is the observed intensity under the Rayleigh (or Compton) peak; β_γa_is the correction factor for absorption of gamma rays in the air present between the target and the detector; β_γt_ is the self-absorption correction factor for the scattered gamma rays in the target; and ɛ_γ_ is the photo-peak efficiency of the gamma detector for Rayleigh (or Compton) scattered gamma rays. The self-absorption correction factor (in reflection geometry) in the target having thickness t is evaluated as follows.

9 $${\text{β}}_{\text{γt}}\text{ = }\frac{1-{\text{e}}^{-\left(\frac{\text{μi}+\text{μe}}{\cos\text{θ}}\right)\text{t}}}{\left(\frac{\text{μi}+\text{μe}}{\cos\text{θ}}\right)\text{t}}$$

Where μ_i_ and μ_e_are the attenuation coefficients for the incident and the scattered radiations, respectively. The well-known WinXcom software, using mixture rule, provides the values of these attenuation coefficients for known gamma ray energies and Z-number of the target. It has been estimated that attenuation of incident and scattered gamma rays in the plastic vial thickness comes to be negligible. The contribution of events resulting from bremsstrahlung originating from slowing down of photoelectrons and Compton recoil electrons is estimated on the basis of an experimental technique suggested by Sandhu *et al.*,[10] and is found to be < 1% under the present experimental conditions.

## Results and Discussion

**Figure 3:**
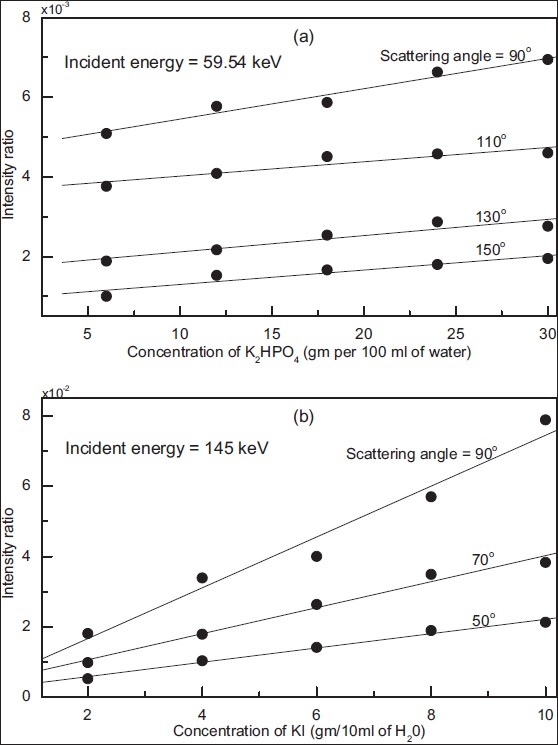
Experimental variation of coherent to incoherent scattered intensity as function of concentration of K_2_HPO_4_ solution (a) simulating mandibular bone and KI solution (b) simulating stable iodine content of thyroid. The measured statistical uncertainties lie within the size of experimental observed points represented by filled circles

The actual values of intensity ratio of Rayleigh to Compton scattering, corrected for various effect discussed above, for different concentration of K_2_HPO_4_ and KI solutions at various selected scattering angles, are given in columns (after column 1) of [Tables Table 1 and Table 2], respectively. The errors quoted in measured intensity ratio indicate percentage error due to statistical uncertainties only. The measured values of Rayleigh to Compton intensity ratio, for various scattering angles, are also plotted as function of concentration of K_2_HPO_4_ (curves of [Figure 3a]) and KI (curves of [Figure 3b]) solution. The solid lines represent the best-fit lines through the experimental data points corresponding to the measured intensity ratios. The equations for the best-fitted regression lines are linear one. The slope of linear curves also increases with scattering angle. This is because an increase in scattering angle results in decrease in the probability for scattering process to occur, and this decrease is more pronounced for Rayleigh scattering in comparison to Compton scattering. The measured values of slope and intercept for the regression lines of phantom simulating thyroid are 0.00217, 0.00323, 0.00178 g^-1^ and 0.00723, 0.0037, and 0.00204 for scattering angles of 50°, 70°, and 90°, respectively.

**Table 1:** Coherent to incoherent intensity ratio, originating from interaction of 59.54 keV incident gamma rays, with phantom at different scattering angles

*Concentration of K_2_HPO_4_ per 100 mL of water*	*Experimental measured values of intensity ratio*			
	
	*90°*	*110°*	*130°*	*150°*
6 g	5.092 (1.24%)	3.767 (1.41%)	1.888 (1.59%)	1.004 (1.89%)
12 g	5.774 (1.18%)	4.090 (1.34%)	2.170 (1.52%)	1.530 (1.44%)
18 g	5.8709 (1.23%)	4.510 (1.31%)	2.5399 (1.42%)	1.666 (1.38%)
24 g	6.635 (1.15%)	4.580 (1.31%)	2.872 (1.32%)	1.802 (1.33%)
30 g	6.948 (1.12%)	4.6027 (1.30%)	2.765 (1.37%)	1.954 (1.38%)

10^-3^ should multiply the numbers. The numbers in brackets indicate percentage error due to statistical uncertainties only.

**Table 2:** Rayleigh to Compton intensity ratio, originating from interaction of 145 keV incident gamma rays with phantom at different scattering angles

*Concentration of KI per 10 mL of water*	*Experimental measured values of intensity ratio*		
	
	*50°*	*70°*	*90°*
2 g	1.807 (1.49)	0.981 (2.14)	0.526 (2.47)
4 g	3.391 (1.03)	1.787 (1.79)	1.033 (1.84)
6 g	4.004 (1.22)	2.634 (1.56)	1.415 (1.55)
8 g	5.698 (0.91)	3.491 (1.43)	1.895 (1.32)
10 g	7.887 (0.74)	3.832 (1.15)	2.128 (1.13)

10^-2^ should multiply the numbers. The numbers in brackets indicate percentage error due to statistical uncertainties only, Figures in parentheses are given in percentage

The measured statistical error in the intensity ratio ranges from 1% to 2.5%. The error in the measurement of photo-peak efficiency of the HPGe detector and self-absorption of gamma rays in the phantom are estimated as < 5% and 1%, respectively. The mandibular bone density and stable iodine content of tissue for a given sample can be determined from the best-fit regression lines of [Figure 3]. Our results show that as concentration of K_2_HPO_4_ and KI solution increases, intensity ratio also increases linearly within experimental estimated error of < 6%.

## Conclusions

The present study fulfils the objective of measurement of mandibular bone density, for either bone resorption studies or as a predictor of osteoporosis, and stable iodine content of tissue using Rayleigh to Compton scattering non-destructive technique. Results indicate that the intensity under the coherent and incoherent peaks observed in scattered gamma ray spectra has the potential to measure mandibular bone density and stable iodine content of tissue, respectively. The use of a phantom to simulate mandibular bone or thyroid iodine, a radioactive source (^241^Am or ^141^Ce) and an HPGe detector provides a non-destructive technique to measure the mandibular bone density and stable iodine content of tissue with a good precision, better than an order of magnitude in the measurement reported by Puumalainen *et al*.[4] The maximum uncertainty in the present measurements is estimated to be <2.5%. No experimental data are available in literature for comparison with the present results at 145 keV incident gamma rays. The present non-destructive measurements provide low-dose inexpensive alternatives to the various other medical techniques. The measurements also do not require absolute source strength of radioactive source and solid angles subtended by the source and detector at the phantom. The present measurements support the work of Morgan *et al*.[1,2] for madibular bone density and of Puumalainen et al.[4] for 59.54 keV gamma rays. The only limitation being half-life of the ^141^Ce source (≈ 30 days) in comparison to 458 years for ^241^Am source. Further studies are required to provide qualitative information on other factors such as ease of use and cost of a complete system. There is also need to measure the dose at the phantom surface for comparison with skin dose received (0.4 mGy) during dental radiography and in other medical diagnostic techniques used for this purpose.
